# Supplementary material for: Thermotolerance in the pathogen Cryptococcus neoformans is linked to antigen masking via mRNA decay-dependent reprogramming
Source: Nat Commun. 2019 Oct 30;10:4950. doi: 10.1038/s41467-019-12907-x (PMC6821889; doi:10.1038/s41467-019-12907-x)
Supplement: Supplementary file 1 — Supplementary Information [file 41467_2019_12907_MOESM1_ESM.pdf]

Supplementary Information

Cryptococcal thermotolerance is linked to antigen masking via mRNA decay-dependent reprogramming

Bloom et al.

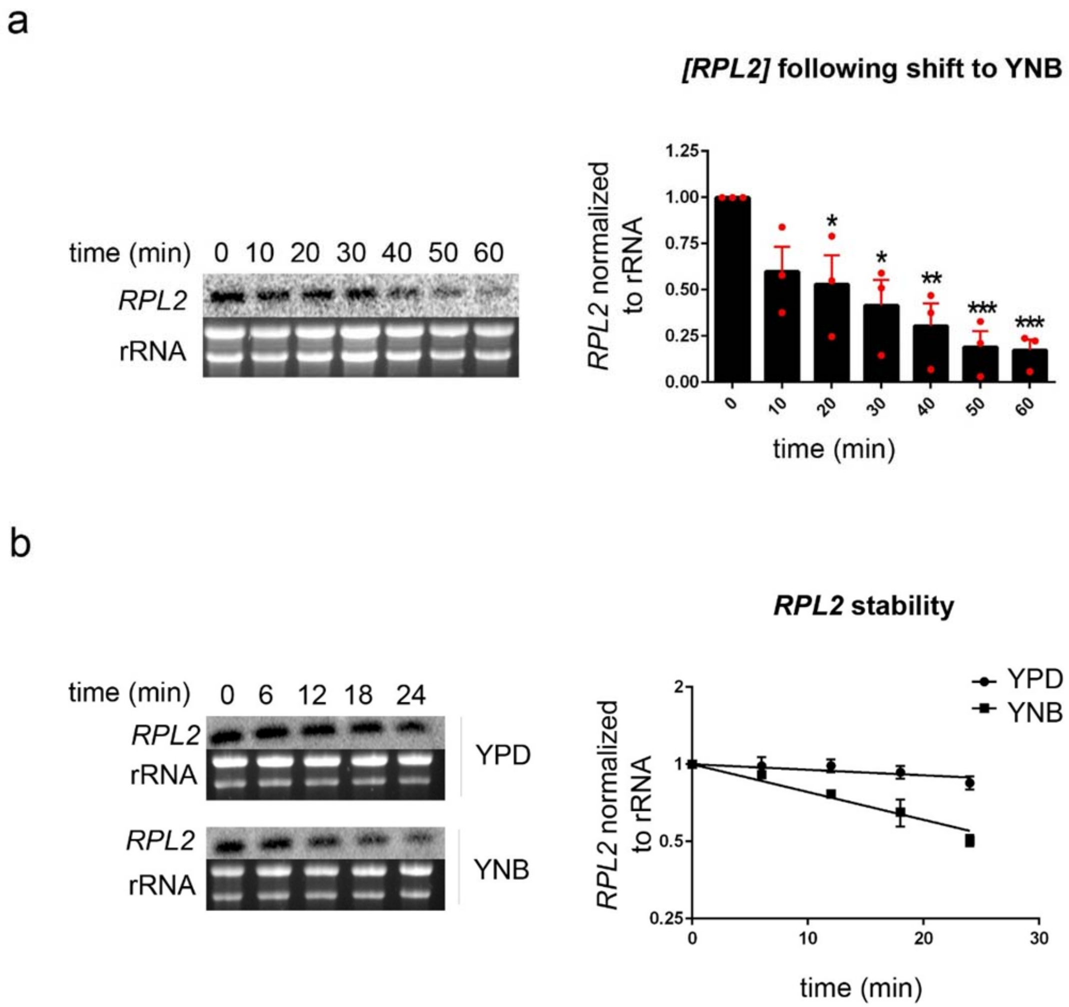

Figure S1. Related to Figure 1. **RP repression and destabilization occurs in response to carbon starvation in *C. amylolentus*. a**. *C. amylolentus* was grown to mid-log phase at 30°C in YPD followed by growth in YNB minimal media at 30□C. RNA from indicated time points was subjected to northern blot analysis for *RPL2*. Graph shows the mean abundance ± SEM of *RPL2* normalized to rRNA (n=3). Expression at each time point was compared to t=0. **b**. To assess stability, the transcriptional inhibitor 1,10-phenanthroline was supplemented in YNB media. RNA from indicated time points was subjected to northern blot analysis for *RPL2*. Mean relative abundance ± SEM of *RPL2* was plotted on a $\log_2$ scale, (n=3).

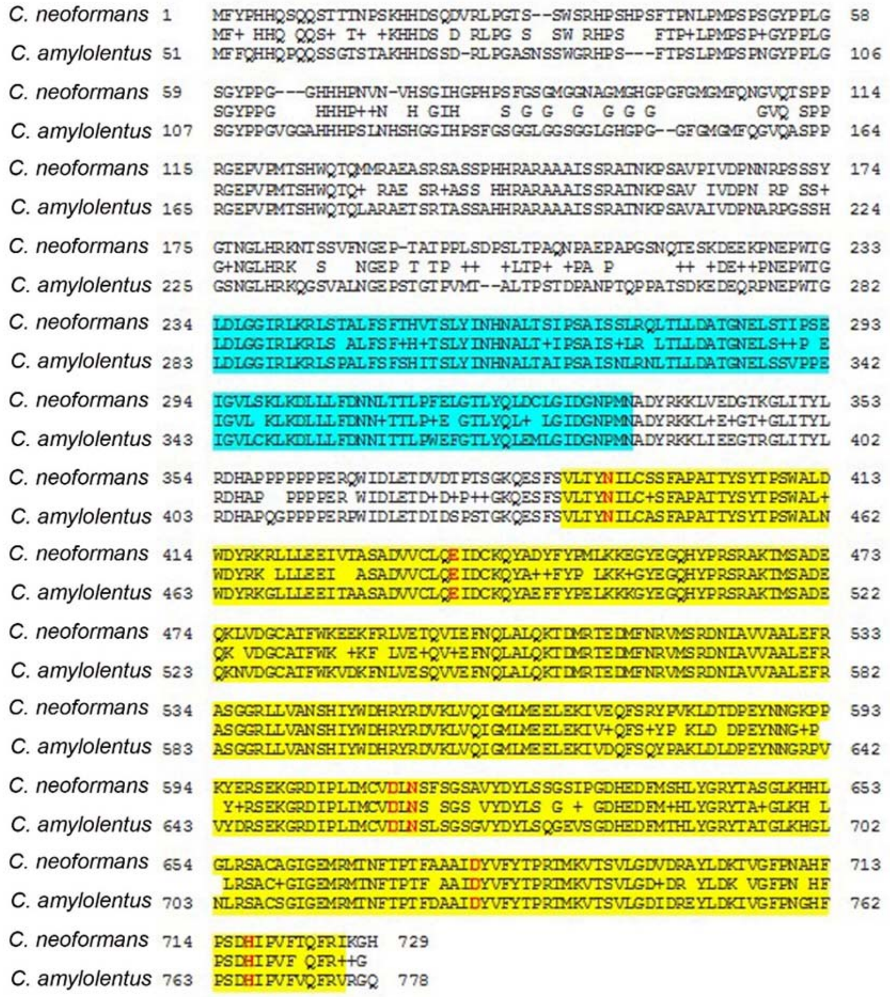

Figure S2. Related to Figure 1. **Protein alignment of Ccr4 homologs.** BLAST analysis using the *C. neoformans* Ccr4 protein sequence as query was used to identify the *C. amylolentus* homolog. The conserved leucine-rich repeat is highlighted in cyan and the catalytic EEP domain is highlighted in yellow. The critical catalytic amino acids for catalytic activity are shown in red.

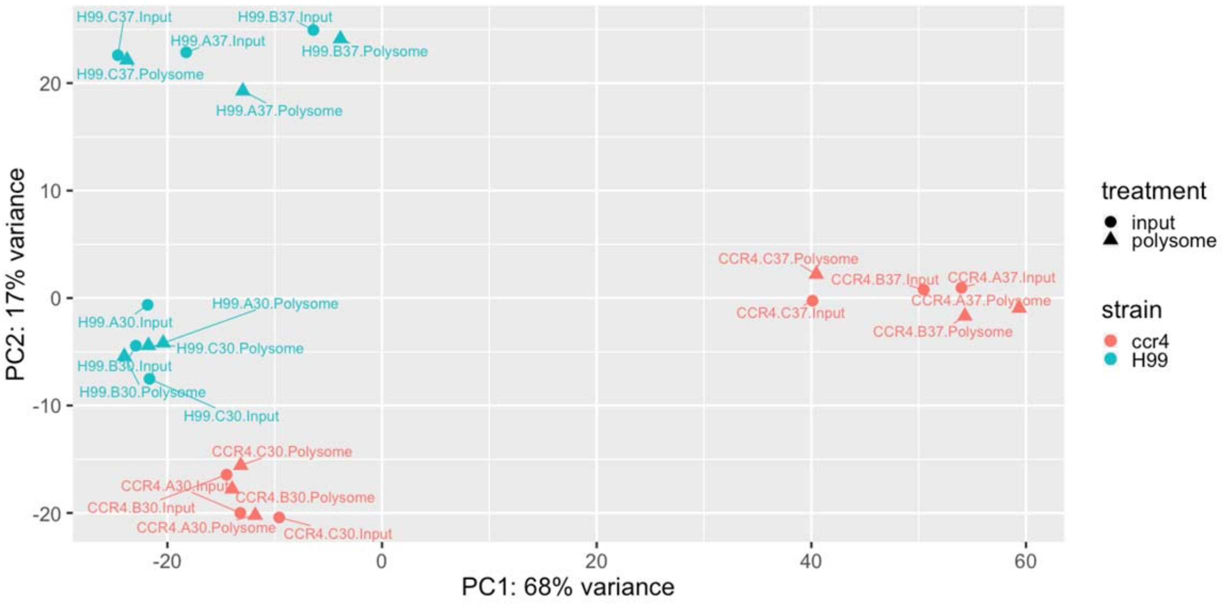

Figure S3. Related to Figure 4. **Principal component analysis for RNA-seq data.** Principal component (PC) analysis was performed for RNA-seq gene expression data acquired from the inputs and translating fractions from the WT and *ccr4*Δ mutant strains during no stress and one hour after a shift to 37°C for three biological replicates.

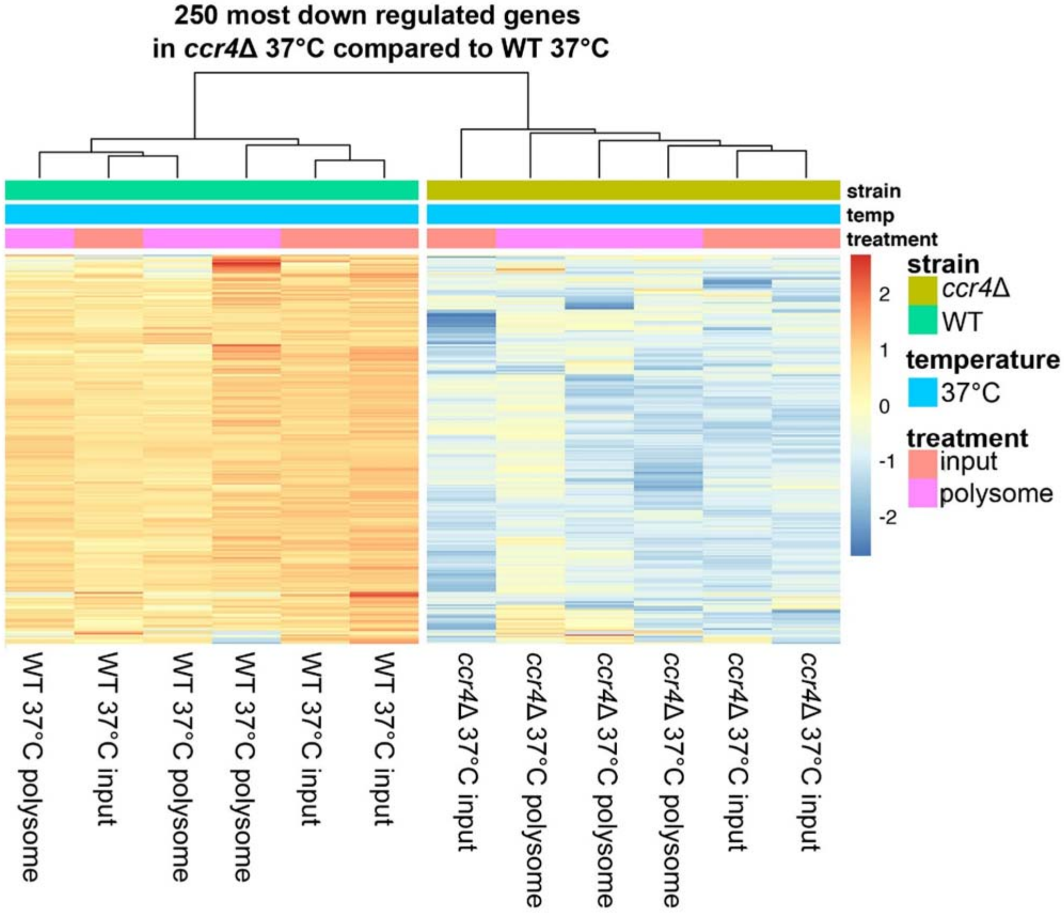

Figure S4. Related to Figure 4. **Genes downregulated in the *ccr4*Δ mutant compared to WT after one hour of 37°C stress.** Heat map comparing 250 genes down regulated in the *ccr4*Δ mutant compared to the WT at 37°C

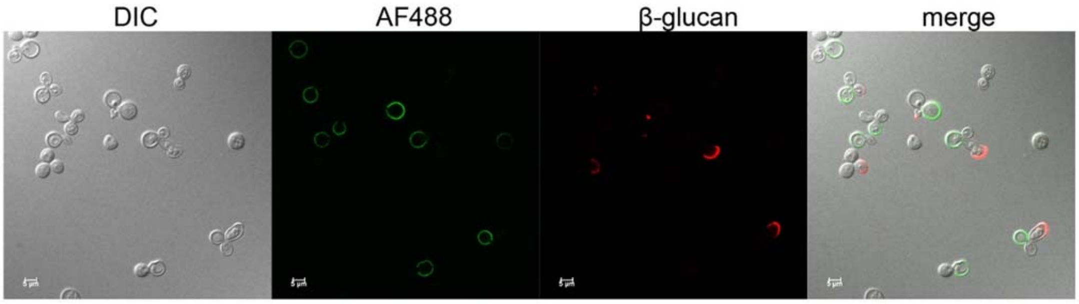

Figure S5. Related to Figure 5. **Mutant cells exposing glucans are newly emerged daughter cells following host-temperature stress.** The *ccr4*Δ mutant was grown at 30°C and stained with Alexafluor-488 (green). Cells were then incubated at 37°C overnight followed by staining for β-(1,3)-glucan (red). Images are representative of 3 biological replicates. Scale bar =5 μm.

Table S1. Related to Figures 4, 5 and 7. **Difference in expression of cell wall remodeling and transcription factor genes in WT at 37°C compared to *ccr4*Δ mutant at 37°C.**

Gene category	Gene accession number	Fold Change RNA-Seq	Fold Change qRT-PCR [a]
Cell wall remodeling	CNAG_03120	-3.87	-2.96 ±0.54
Cell wall remodeling	CNAG_03856	-2.06	-2.10 ±0.22
Cell wall remodeling	CNAG_06898	-2.48	-2.33 ±0.60
Cell wall remodeling	CNAG_05803	-2.88	-4.06 ±2.04
Transcription Factor	CNAG_01431 (*HOB1*)	-6.31	-5.72±1.18
Transcription Factor	CNAG_03115 (*FZC46*)	-23.0	-9.48±1.89
Transcription Factor	CNAG_06188 (*FZC15*)	-1.91	-8.31±4.26

[a]Reported as relative Fold change ±SEM, n=3.

Table S2. Related to Figures 4 and 5. **Cell wall remodeling genes down-regulated in the *ccr4*Δ mutant compared to WT one hour after shift to host-temperature by RNA-Seq.**

Accession number	gene description	log2 difference
CNAG_03326	chitin synthase 2	-3.047921231
CNAG_06487	chitin synthase 6	-2.53191701
CNAG_02351	conserved hypothetical protein	-2.045210384
CNAG_03120	alpha-1,3-glucan synthase	-1.952786754
CNAG_05138	exo-beta-1,3-glucanase	-1.917029697
CNAG_00699	transmembrane receptor	-1.830161471
CNAG_05663	cell wall integrity protein scw1	-1.542967672
CNAG_05803	exo-beta-1,3-glucanase	-1.525888398
CNAG_05818	chitin synthase 5	-1.500068256
CNAG_01941	glucan synthesis regulatory protein	-1.457146095
CNAG_06898	chitin synthase export chaperone	-1.31068817
CNAG_05778	WSC domain-containing protein	-1.302075795
CNAG_00546	chitin synthase 6	-1.28959723
CNAG_06508	glucan synthase	-1.276533716
CNAG_02850	glucan endo-1,3-alpha-glucosidase agn1	-1.129427265
CNAG_04874	conserved hypothetical protein	-1.113714142
CNAG_03856	conserved hypothetical protein	-1.045489176
CNAG_07499	chitin synthase 8	-1.006751613
CNAG_05080	calcofluor white hypersensitive protein	-0.998129006
CNAG_06031	beta-glucan synthesis-associated protein	-0.944208079
CNAG_06726	chitin synthase regulator 3	-0.818079972
CNAG_05581	chitin synthase 4	-0.816544394
CNAG_00475	WSC domain-containing protein	-0.874484819

***Table S3.* Primers used in this study.**

Primer name	Oligonucleotide
F-CamyloCCR4	TAATAACAATTGATGCCCGTGTGCTGGCGG
R-CamyloCCR4	TAATAATCTAGATTATTGCTGACCCCTGACTCGG
F-CneoCCR4-up	TAATAAGGTACCCTGCTGTTTCAACTCCATAGGC
R-CneoCCR4-up	TAATAACAATTGTGGCTGTGGTGTGAGCGT
F-CneoCCR4-down	TAATAATCTAGAGACATCTTTCCATCCATTCC
R-CneoCCR4-down	TAATAAGGTACCGGAATAGTTTGACGGGTGG
RPL2 Forward	ATGGGTCGAGTCATCCGCGC
RPL2 Reverse	TTAAGCGTTGTCGACGTTCTTGC
F-CNAG_03115 (FZC46)	GTATGAGCTGGCCACTTCTC
R-CNAG_03115 (FZC46)	CCCACGAACAACCTTCATCATCC
F-CNAG_01430 (HOB1)	AGCTTCGGGATGGCTACAAATAAC
R-CNAG_01430 (HOB1)	TCCTTCCCATGGCTTCGTTGTC
F-CNAG_06188 (FZC15)	GGTACTCGTCAACTATTTCCACAGCC
R-CNAG_06188 (FZC15)	GGATCGCGAATCGCTCAGAGG
F-CNAG_06688 (mitofusin)	CCTGGATCTTCCTCACCG
R-CNAG_06688(mitofusin)	CAGGTGCAACTGAGAGCG
F-CNAG_03120	ATGCCTGGTTGGTGGTTCC
R-CNAG_03120	ATTCGACAACGGGGAAACG
F-CNAG_03856	AACGTCCCTGGAACCCTTGC
R-CNAG_03856	CATCAGCCCTTCGTCGGAGG
F-CNAG_06898	CAAGGTCTACCCTTGGTTCG
R-CNAG_06896	GATCAAGACACACACACAAGG
F-CNAG_05803	ACCCAGATGAGTATGACGC
R-CNAG_05803	GCCAGAACCACTACATTGG
F-CNAG_03297	GGTTCATCTGACAACACCATTGC
R-CNAG_03297	CATCATCGGGAGTTTGTTCAGC
