# Supplementary material for: Thermotolerance in the pathogen Cryptococcus neoformans is linked to antigen masking via mRNA decay-dependent reprogramming
Source: Nat Commun. 2019 Oct 30;10:4950. doi: 10.1038/s41467-019-12907-x (PMC6821889; doi:10.1038/s41467-019-12907-x)
Supplement: Supplementary file 3 — Description of Additional Supplementary Files [file 41467_2019_12907_MOESM3_ESM.pdf]

Description of Additional Supplementary Files

File Name: Supplementary Data 1

Description: Summary of All Differentially Expressed Genes fromt the RNA-seq experiments.

File Name: Supplementary Data 2

Description: Transcription factors found down-regulated in the *ccr4*Δ polysomes and input samples at 37°C
