# Supplementary material for: Thermotolerance in the pathogen Cryptococcus neoformans is linked to antigen masking via mRNA decay-dependent reprogramming
Source: Nat Commun. 2019 Oct 30;10:4950. doi: 10.1038/s41467-019-12907-x (PMC6821889; doi:10.1038/s41467-019-12907-x)
Supplement: Supplementary file 6 — Reporting Summary [file 41467_2019_12907_MOESM6_ESM.pdf]

nature research

Corresponding author(s): John C. Panepinto

Reporting Summary

Nature Research wishes to improve the reproducibility of the work that we publish. This form provides structure for consistency and transparency in reporting. For further information on Nature Research policies, see Authors & Referees and the Editorial Policy Checklist.

Statistical parameters

When statistical analyses are reported, confirm that the following items are present in the relevant location (e.g. figure legend, table legend, main text, or Methods section).

n/a	Confirmed	
☐	☒	The exact sample size (n) for each experimental group/condition, given as a discrete number and unit of measurement
☐	☒	An indication of whether measurements were taken from distinct samples or whether the same sample was measured repeatedly
☐	☒	The statistical test(s) used AND whether they are one- or two-sided<br>*Only common tests should be described solely by name; describe more complex techniques in the Methods section.*
☐	☒	A description of all covariates tested
☐	☒	A description of any assumptions or corrections, such as tests of normality and adjustment for multiple comparisons
☐	☒	A full description of the statistics including central tendency (e.g. means) or other basic estimates (e.g. regression coefficient) AND variation (e.g. standard deviation) or associated estimates of uncertainty (e.g. confidence intervals)
☐	☒	For null hypothesis testing, the test statistic (e.g. F, t, r) with confidence intervals, effect sizes, degrees of freedom and P value noted<br>*Give P values as exact values whenever suitable.*
☒	☐	For Bayesian analysis, information on the choice of priors and Markov chain Monte Carlo settings
☒	☐	For hierarchical and complex designs, identification of the appropriate level for tests and full reporting of outcomes
☒	☐	Estimates of effect sizes (e.g. Cohen's d, Pearson's r), indicating how they were calculated
☐	☒	Clearly defined error bars<br>*State explicitly what error bars represent (e.g. SD, SE, CI)*

Our web collection on statistics for biologists may be useful.

Software and code

Policy information about availability of computer code

Data collection	For RNA-seq basecalling files were converted to FASTQ format using Illumina's bcl2fastq version 2.17.1.14<br><br>BD FACSDIVA was used to collect flow cytometry data.
Data analysis	For RNA-seq analyses the following software was used: Quality of Sequencing: FastQC version 0.11.5 and Fastq_screen version 0.11.1 were used for quality of sequencing, read trimming was performed using cutadapt version 1.16, alignment was performed using hisat2 version 2.1.0, compression and sorting was performed with samtools version 1.7, quantification of reads was done with Subread featureCounts version 1.6.0, and differential expression was detected with Bioconductor's DESeq2 version 1.20.0.<br>GraphPad Prism version 6.05 was used for statistical analyses. FlowJo version 10.0.8 was used to analyze flow cytometry data.

For manuscripts utilizing custom algorithms or software that are central to the research but not yet described in published literature, software must be made available to editors/reviewers upon request. We strongly encourage code deposition in a community repository (e.g. GitHub). See the Nature Research guidelines for submitting code & software for further information.

Data

Policy information about availability of data

All manuscripts must include a data availability statement. This statement should provide the following information, where applicable:

- Accession codes, unique identifiers, or web links for publicly available datasets
- A list of figures that have associated raw data
- A description of any restrictions on data availability

RNA-sequencing data that support the findings in this study have been deposited in GEO under code GSE121183.

Field-specific reporting

Please select the best fit for your research. If you are not sure, read the appropriate sections before making your selection.

☒ Life sciences ☐ Behavioural & social sciences ☐ Ecological, evolutionary & environmental sciences

For a reference copy of the document with all sections, see nature.com/authors/policies/ReportingSummary-flat.pdf

Life sciences study design

All studies must disclose on these points even when the disclosure is negative.

Sample size	For analysis of macrophage association with yeast three mice per yeast strain were used for each experiment, and the experiment was performed 3 times. No sample size calculation was performed.
Data exclusions	No data were excluded from our analyses.
Replication	For each of our experiments, reproducibility was ensured by performing at least 3 biological replicates.
Randomization	Upon arrival mice were randomly caged by our animal facilities staff.
Blinding	Blinding was not used.

Reporting for specific materials, systems and methods

Materials & experimental systems

n/a	Involved in the study	
☒	☐	Unique biological materials
☐	☒	Antibodies
☒	☐	Eukaryotic cell lines
☒	☐	Palaeontology
☐	☒	Animals and other organisms
☒	☐	Human research participants

Methods

n/a	Involved in the study	
☒	☐	ChIP-seq
☐	☒	Flow cytometry
☒	☐	MRI-based neuroimaging

Antibodies

Antibodies used

Mouse IgM MOPC-104E, Sigma, Cat#: M5909
Mouse anti-beta-(1,3)-glucan antibody, BioSupplies Australia, Cat#: 400-2
Donkey anti-mouse IgG Alexafluor-488, Invitrogen, ThermoFisherScientific, Cat# :A-21202
Mouse anti-human IgG1 Alexafluor-488 Invitrogen, ThermoFisherScientific, Cat#: A-10631
Goat anti-mouse IgM (μ chain) Alexafluor-488, Invitrogen, ThermoFisherScientific, Cat#: A-21042
Rat anti-CD11b-BV605 clone M1/70, BD Horizon, Cat# 563015
Mouse anti-CD45 monoclonal Antibody, APC-eFluor 780 clone 30-F11, ebioscience, ThermoFisher Scientific, Cat# 47-0451-80, RRID AB_1548790
Rat anti-MerTK, clone 2B10C45, Biolegend ,Cat# 151506; RRID AB_2617037
Hamster anti-CD11c clone N418 eBioscience, ThermoFisher Scientific, Cat# 14-0114-81, RRID AB_467114

Validation

Commercial antibodies were tested by suppliers. Additionally, studies in C. neoformans and other fungal organisms have

demonstrated the ability of the cell wall antibodies to recognize these constituents.

Animals and other organisms

Policy information about studies involving animals; ARRIVE guidelines recommended for reporting animal research

Laboratory animals	All mice used in this study were BALBc/J, female, and 5-6 weeks old.
Wild animals	This study did not involve wild animals.
Field-collected samples	This study did not involve field-collected samples.

Flow Cytometry

Plots

Confirm that:

☒ The axis labels state the marker and fluorochrome used (e.g. CD4-FITC).

☒ The axis scales are clearly visible. Include numbers along axes only for bottom left plot of group (a 'group' is an analysis of identical markers).

☒ All plots are contour plots with outliers or pseudocolor plots.

☒ A numerical value for number of cells or percentage (with statistics) is provided.

Methodology

Sample preparation	To obtain BAL, lungs were lavaged with 1.5 mL sterile PBS three times using a 22 gauge blunt-end needle placed in the trachea. Red blood cells were lysed in ACK lysing buffer (Lonza). For isolation of lung tissue leukocytes, mice were subsequently transcardially perfused with PBS. Lungs were harvested and minced in digestion media. Tissues were digested at 37˚C for 45 minutes and sequentially passed through a 70µm and 40µm filter. Red blood cells were lysed in ACK lysing buffer. Single-cell suspensions were stained with HBSS containing extracellular antibody stains and Live/Dead Fixable Aqua dead cell stain (Life Technologies).
Instrument	BD LSRFortessa
Software	Data was collected with BD FACSDIVA and analyzed using FlowJo version 10.0.8.
Cell population abundance	10,000 cells
Gating strategy	Live/Dead (live+), CD45+, MerTK+, Crypto+

☒ Tick this box to confirm that a figure exemplifying the gating strategy is provided in the Supplementary Information.
